# Aureusimines in *Staphylococcus aureus* Are Not Involved in Virulence

**DOI:** 10.1371/journal.pone.0015703

**Published:** 2010-12-29

**Authors:** Fei Sun, Hoonsik Cho, Do-Won Jeong, Chunling Li, Chuan He, Taeok Bae

**Affiliations:** 1 Department of Chemistry and Institute for Biophysical Dynamics, University of Chicago, Chicago, Illinois, United States of America; 2 Department of Microbiology and Immunology, Indiana University School of Medicine-Northwest, Gary, Indiana, United States of America; National Institutes of Health, United States of America

## Abstract

**Background:**

Recently, dipeptide aureusimines were reported to activate expression of staphylococcal virulence genes, such as alpha-hemolysin, and increase *S. aureus* virulence. Surprisingly, most of the virulence genes affected by aureusimines form part of the regulon of the SaeRS two component system (TCS), raising the possibility that SaeRS might be directly or indirectly involved in the aureusimine-dependent signaling process.

**Methodology/Principal Findings:**

Using HPLC analyses, we confirmed that a transposon mutant of *ausA*, the gene encoding the aureusimine dipeptide synthesis enzyme, does not produce dipeptides. However, the transposon mutant showed normal hemolysis activity and alpha-hemolysin/SaeP production. Furthermore, the P1 promoter of the *sae* operon, one of the targets of the SaeRS TCS, showed normal transcription activity. Moreover, in contrast to the original report, the *ausA* transposon mutant did not exhibit attenuated virulence in an animal infection model. DNA sequencing revealed that the *ausA* deletion mutant used in the original study has an 83 nt-duplication in *saeS*. Hemolysis activity of the original mutant was restored by a plasmid carrying the *sae* operon. A mutant of the *sae* operon showed elevated resistance to chloramphenicol and erythromycin, two antibiotics widely used during staphylococcal mutagenesis. At 43°C in the presence of erythromycin and aeration, the conditions typically employed for staphylococcal mutagenesis, an *saeR* transposon mutant grew much faster than a control mutant and the *saeR* mutant was highly enriched in a mixed culture experiment.

**Conclusions/Significance:**

Our results show that the previously reported roles of aureusimines in staphylococcal gene regulation and virulence were due to an unintended mutation in *saeS*, which was likely selected due to elevated resistance of the mutant to environmental stresses. Thus, there is no evidence indicating that the dipeptide aureusimines play a role in *sae*-mediated virulence factor production or contribute to staphylococcal virulence.

## Introduction

*Staphylococcus aureus* is an important human pathogen that causes a wide variety of diseases, ranging from minor skin infections to life-threatening blood infections, endocarditis, pneumonia, and toxic shock syndrome [1], [2]. The bacterial pathogen can infect almost every part of the human body and this versatility of the bacterium is, at least partly, due to numerous virulence factors such as hemolysins, leukocidins, and immune modulators. The expression of these virulence factors is coordinately regulated, for example by multiple transcription factors or two component systems [3]. The SaeRS two component system (TCS) in particular plays a key role in the production of important virulence factors such as alpha-hemolysin, coagulase, and fibronectin binding proteins [4], [5], [6], [7], [8] in vitro and it has been shown that SaeRS is essential for in-vivo production of alpha-hemolysin [9].

**Figure 1:**
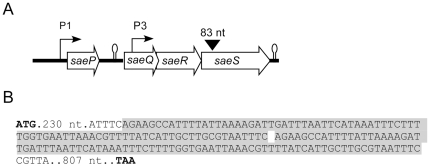
The *sae* locus of *S. aureus* (A) and *saeS* gene in the original *ausA* deletion mutant (B). A. The four open reading frames in the *sae* operon are shown as open arrows with the corresponding gene names. The two *sae* promoters, P1 and P3, are indicated with the arrows pointing out the transcription direction. The 83 nt duplication found in *ausA* deletion mutant is shown as a reversed triangle. Putative transcription terminators are indicated by stem-loop structures. **B**. Chromosomal DNA was purified from the original *ausA* deletion mutant; then the s*aeRS* region was PCR-amplified and sequenced. The 83 nt duplicated in *saeS* is indicated by a gray-colored shadow. The start and stop codons are bold faced. For clarity, only numbers are shown for intervening nucleotides.

The SaeRS TCS is encoded by the *saePQRS* operon, in which *saeR* and *saeS* encode the response regulator and sensor kinase, respectively (Fig. 1A). The *sae* operon has two promoters, P1 and P3 [10], [11], [12]. Since the P1 promoter is positively autoregulated by the SaeRS system [11], [13], [14], along with alpha-hemolysin production, P1 promoter activity and P1-driven production of SaeP are good indicators for the function of SaeRS. In strain Newman, a clinical isolate widely used in staphylococcal research, SaeS has an L18P mutation in the first transmembrane domain, which renders this sensor kinase constitutively active [10].

**Figure 2:**
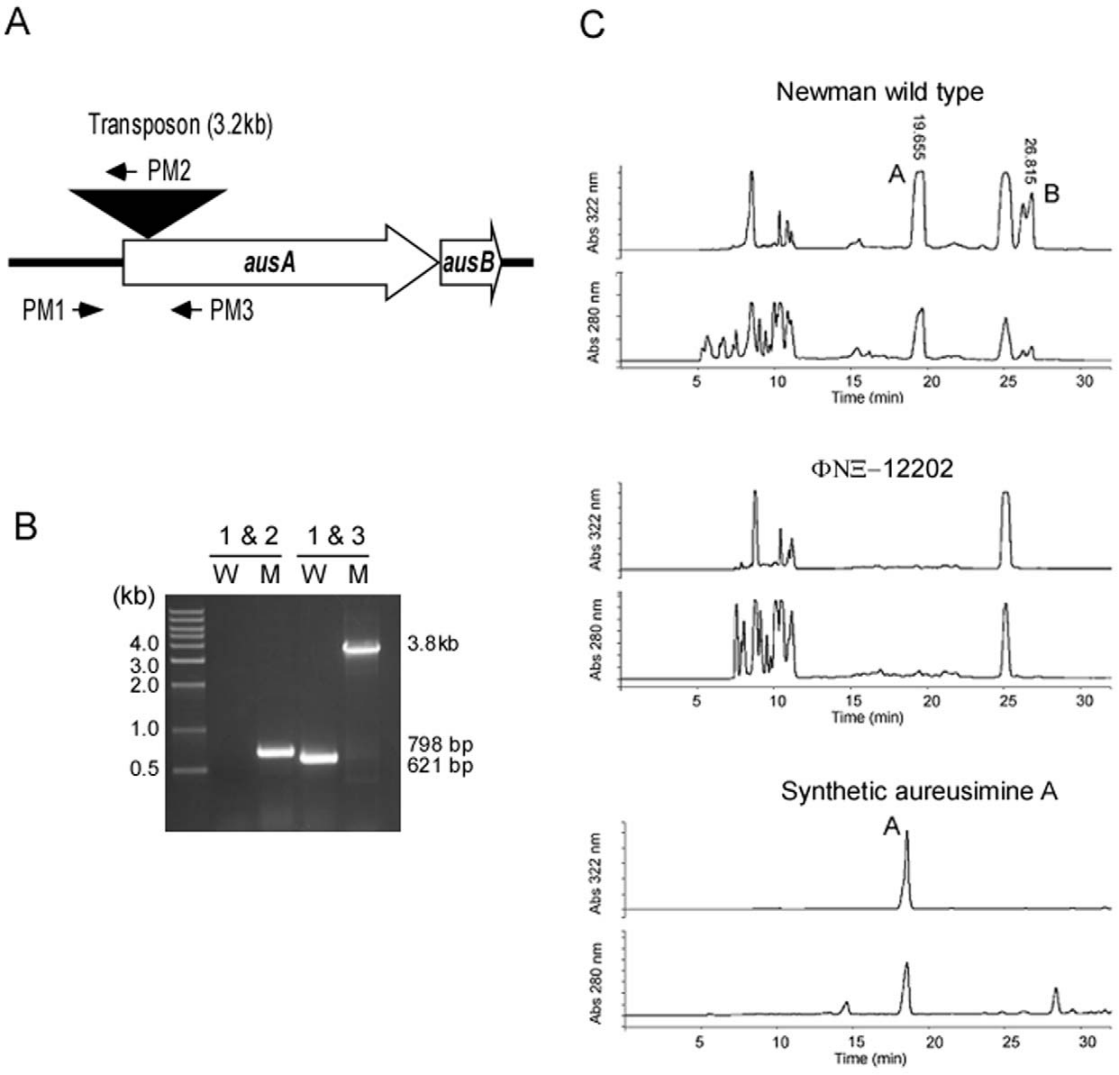
The *ausA* transposon mutant ΦΝΞ-12202 does not produce aureusimines. **A**. The *ausAB* operon in the transposon mutant ΦΝΞ-12202. The transposon insertion is indicated by a reversed triangle. The primers (PM1, PM2, and PM3), used for PCR-amplification, are shown as arrows. **B**. PCR amplification of the *ausA* region of wild-type Newman (W) and the transposon insertion mutant ΦΝΞ-12202 (M). The numbers at the top represent the primer pairs. The calculated size of each product is indicated on the right of the picture. Molecular size markers are shown to the left. **C**. The absence of aureusimines in the culture supernatant of the transposon mutant ΦΝΞ-12202. The culture supernatants from wild-type Newman and the transposon mutant were extracted with ethyl acetate and suspended in methanol. The supernatant extracts were analyzed with HPLC. As a control, synthetic aureusimine A was used. A, aureusimine A; B, aureusimine B.

Recently, nonribosomally synthesized dipeptides, named aureusimine A and B, were reported by Wyatt et al. to increase staphylococcal virulence by activating the expression of virulence genes, including those encoding alpha-hemolysin, coagulase, and fibronectin binding proteins [15]. The dipeptides are synthesized by the products of two genes, *ausA* (NWMN_0123) and *ausB* (NWMN_0124) (Fig. 2A). In the report by Wyatt et al., the *ausA* deletion mutant of strain Newman showed reduced hemolysis on blood agar and attenuated virulence in a murine abscess formation model [15]. Intriguingly, almost all (39 out of 40) virulence genes reported to be affected by the *ausA* mutation in that study are known to be regulated by the SaeRS TCS [15]. In particular, activities of the *hla* (alpha-hemolysin) and *sae* P1 promoters were severely reduced, suggesting that the SaeRS TCS is not functional in the *ausA* mutant. This apparent convergence of regulons implies that aureusimines might be the activation ligands for SaeS. However, since the SaeS sensor kinase is constitutively active in strain Newman [10], exactly how the *ausA* mutation (i.e., the absence of the dipeptides) could abolish SaeRS-dependent signaling is puzzling. The aim of the present study was to examine whether the aureusimine dipeptides are the activation signals for SaeRS. In the process of addressing this question, we found that the original *ausA* mutant contains an inadvertent mutation in *saeS* and, therefore, the observed involvement of aureusimines in staphylococcal virulence was due to that mutation in *saeS*.

## Materials and Methods

### Ethics Statement

The animal study presented in this report (Approval ID: NW-20) was approved by the Institutional Animal Care and Use Committee at Indiana University School of Medicine-Northwest, which complies with the guidelines of the National Institutes of Health.

#### Bacterial strains, plasmids and culture conditions

The bacterial strains used in this study are listed in Table 1. *Staphylococcus aureus* cells were grown in tryptic soy broth (TSB). For routine culture of transposon mutants, erythromycin (10 µg/ml) was added, while chloramphenicol (5 µg/ml) was added to cells containing the *lacZ* reporter plasmid pCL-P1-lacZ [16].

**Table 1:** Bacterial strains and plasmids used in this study.

Strain/Plasmid	Relevant Characteristics	Source
***E.coli***		
DH5α		Stratagene
***S. aureus***		
RN4220	restriction deficient, prophage-cured	[39]
Newman	a clinical isolate, L18P substitution in SaeS	[40]
ΦΝΞ-12202	strain Newman with the *bursa aurealis* transposon insertion in *ausA*	Phoenix library
ΦΝΞ-11568	strain Newman with the *bursa aurealis* transposon insertion in *hla*	Phoenix library
ΦΝΞ-9654	strain Newman with Tn917 insertion in *saeP*	Phoenix library
NMΔ*sae*	strain Newman with deletion of the *sae* operon	This study
NewmanΔ*ausA*	strain Newman with *ausA* replaced by *ermB,* Em^r^	[15]
NM::*saeR*	strain Newman with the *bursa aurealis* transposon insertion in *saeR* ( = ΦΝΞ-01594),Em^r^	Phoenix library
NM::*geh*	strain Newman with the *bursa aurealis* transposon insertion in *geh* ( = ΦΝΞ-0040), Em^r^	Phoenix library
**Plasmids**		
pCL55	single-copy integration plasmid, Cm^r^	{Lee, 1991#978}
pCL55-sae	pCL55 carrying s*aePQRS* operon	this study
pCL-P1-lacZ	pCL55 carrying P1-*lacZ* reporter gene	[16]

Em^r^: resistant to erythromycin; Cm^r^, resistant to chloramphenicol.

#### Generation of *sae*-deletion mutant (NMΔsae)

The *sae* operon was deleted from the genome of *S. aureus* strain Newman by the allelic replacement plasmid pKOR1 as reported previously [17]. For the deletion, flanking DNA fragments (1 kb each) were PCR-amplified from the genome with the following primers: P667 (5′- GGGG ACA AGT TTGTACAAAA AAGCAGGCT G GGGAAGTCA TTACACAAAC ACATC-3′)/P668 (5′- GTA GGATCC CACAA ATTAGACATTACGTCATAATCC-3′) for *saeP*-flanking DNA, and P669 (5′- ATA GGATCC ATTCATGCTAACTCCTCATT TCTTC-3′)/P670 (5′-GGGGACC ACTTTGTACAAGAAAGCTGGGT GTT ATGCAAAGTAATGATATGAATCAC-3′) for s*aeS* -flanking DNA. For the DNA amplification, the enzyme Phusion (New England Biolabs) was used.

### Chemical synthesis of aureusimine A

The synthesis of aureusimine A was adapted from the previously reported synthesis of Phevalin 2 [18]. Details of the synthesis can be found in Supporting Information (Fig. S1).

### HPLC analysis of culture supernatants

Preparation and HPLC analysis of organic cell extracts followed the procedures described by Wyatt et al. [15] with a few modifications. Briefly, overnight cultures of *S. aureus* Newman and the transposon mutant of *ausA* (ΦΝΞ-12202) were diluted 1∶100 into 1 L of fresh TSB containing nalidixic acid (10 µg/ml) and grown for 3 days on a rotary shaker (37°C, 175 rpm). The cells and broth were extracted with 1 L of ethyl acetate twice, then evaporated and dissolved in 1 ml of methanol. Aliquots of 0.25 ml of the concentrated extract were analyzed by HPLC with a C18 column (Higgins Analytical Proto 200). The flow rate of the mobile phase was 3 ml/min. Compounds were eluted using a linear gradient from 20% to 80% acetonitrile in 0.1% TFA over 40 min. Eluted compounds were detected by UV absorbance at 280 nm and 322 nm. In the analysis, aureusimine A and B eluted at 19.66 min and 26.82 min, respectively.

#### PCR characterization of the transposon mutant

The wild-type Newman and the transposon mutant ΦΝΞ-12202 strains were grown in 3 ml of TSB at 37°C overnight, and chromosomal DNAs were purified with a Zippy DNA purification kit (Zymo Research). To detect the transposon insertion, the primers PM1 (5′-TTTTAACAATTGGTGCTAGCATGC-3′) and PM2 (5′-TTTATGGTA CCATTTCATTTTCCTGCTTTTTC-3′) were used. To amplify the 5′ part of *ausA*, we used the primers PM1 and PM3 (5′-TTAGC GAGTTTAACC CAATACGC-3′). The primers were annealed to chromosomal DNA at 55°C for 15 seconds, and the DNA was amplified by the enzyme Phusion (New England Biolabs) at 72°C for 1 min with 30 cycles.

#### Construction of plasmid pCL55-sae

The entire *saePQRS* operon was PCR-amplified from the genome of *S. aureus* strain Newman using Phusion polymerase (New England Biolabs) with the primers: P671 (5′- AAC GAATTC TTGGTACTTGTATTTAATCGTCTATC-3′)/P783 (5′-AAACTT CCGCGG TTATGACGTAATGTCTAATTTGTG-3′). The PCR product was cut with *Eco*RI and ligated into the single-copy integration plasmid pCL55 [19] cut with *Eco*RI and *Sma*I. The ligated plasmid was transformed into *Escherichia coli* DH5α. To confirm that no mutation was introduced during PCR amplification, the resulting plasmid pCL55-sae was sequenced; no mutation was found in the cloned *saePQRS* operon.

#### Cell fractionation and Western blot hybridization

Cells from an overnight culture were diluted in TSB containing appropriate antibiotics, and grown with shaking at 37°C to the stationary growth phase (OD_600_ = 6). All cultures were normalized by measuring optical density (OD_600_ = 0.6). Supernatant and cells were separated by centrifugation. Proteins in the culture supernatants were precipitated by trichloroacetic acid (10% final concentration) on ice for 20 min, washed with 1 ml acetone, and then air-dried. The dried proteins were suspended in 20 µl 1× SDS-PAGE sample buffer (40 mM Tris HCl, pH 6.8, 2% SDS, 2 mM beta-mercaptoethanol, 4% glycerol, 0.01% bromophenol blue). Cells collected by centrifugation were suspended in 50 µl of Tris HCl (pH 8.0); then 2 µl lysostaphin (2 mg/ml) was added. After incubation at 37°C for 30 min, 50 µl of 2× SDS-PAGE sample buffer was added. The supernatant sample was used to detect alpha-hemolysin, while the cell pellet sample was used to detect SaeP. The samples were separated by 15% SDS-PAGE and the proteins were transferred onto a nitrocellulose membrane (0.45 µm, Whatman). The membrane was blocked with 10% skim milk and incubated with primary antibody (either alpha-hemolysin antibody from rabbit or SaeP antibody from chicken) for 1 h at room temperature; then the blot was incubated with horseradish peroxidase (HRP)-conjugated secondary antibody (either anti-rabbit IgG or anti-chicken IgG). Signals were detected by a luminal enhancer solution detection kit (Thermo). The alpha-hemolysin antibody was purchased from Sigma-Aldrich. The SaeP antibody was generated by Genscript.

#### LacZ assay

Cells carrying the plasmid pCL-P1-lacZ [16] were grown overnight and diluted in fresh media containing chloramphenicol (5 µg/ml). The cells were further grown with shaking at 37°C to mid-log phase (OD_600_ = 0.8). Then cells were harvested, washed with AB buffer (60 mM K_2_HPO_4_, 40 mM KHPO_4_, 100 mM NaCl, pH 7.0), and suspended in 100 µl of the same buffer. After 5 µl lysostaphin (2 mg/ml) was added, the cell suspension was incubated for 15 min at 37°C and then mixed with 900 µl of AB buffer containing 0.1% (v/v) Triton X-100. The β-galactosidase assay was performed at room temperature. As a substrate, 4-methyl umbelliferyl β-D galactopyranoside (MUG, Sigma) was used in the hydrolysis reaction, which was read at 366 nm excitation and 445 nm emission wavelengths.

#### Murine model of abscess formation

The effect of the *ausA* mutation on staphylococcal virulence was examined as described previously [20]. Briefly, after the weight of the mice was measured, 1×10^7^ cfu (colony forming unit) of Newman wild type and the *ausA* transposon mutant ΦΝΞ-12202 were administered to 10 Balb/c mice (Harlan, USA) via retro-orbital injection. Four days after the injection, the mice were sacrificed and their organs (heart, kidney, liver, and spleen) were harvested. The harvested organs were homogenized; then the cfu of bacteria in the organs was measured using serial dilutions on TSA plates.

#### Antibiotic resistance test

Antibiotic resistances of the *sae* deletion mutant and the wild-type Newman strains were compared by measuring growth with Bioscreen C and EZExperiment software (Growth Curves USA). In brief, approximately 2×10^5^ cfu of bacteria was inoculated into 200 µl of TSB containing a test antibiotic and incubated for 24 h without shaking at either 37°C or 43°C. The highest concentration of the test antibiotic was 10 µg/ml, which was serially diluted using 1∶2 dilution steps. For data stability, the bacterial growth was measured by the optical density (OD) at 420 nm–580 nm. The presented data are the mean of quadruplicate measurements.

#### Environmental effects on growth

The transposon insertion mutants of *geh* (glycerol ester hydrolase) and s*aeR* were grown in 3 ml TSB containing 10 µg/ml of erythromycin (TSB_erm10_) at 37°C overnight. The next day, cells were collected by centrifugation and suspended in phosphate-buffered saline (PBS) to OD_600_ = 1. The cell suspension (500 µl) was inoculated into 500 ml TSB and incubated under various conditions (37°C vs. 43°C, +/− erythromycin, +/− aeration at 250 rpm). Cell growth was determined over 24 h by measuring OD_600_.

#### Enrichment test

The transposon insertion mutants of *geh* and s*aeR* were prepared as described above. The cell suspensions (1.5 ml each) were mixed and 500 µl of the mixed culture was inoculated into 500 ml TSB either with or without erythromycin (10 µg/ml). The cultures were grown at 43°C for 16 h with shaking (250 rpm). Chromosomal DNA was purified from 1 ml of the initial mixed culture (input) and the final cultures either with or without erythromycin. The changes of each strain in the final cultures were analyzed by quantitative real time PCR (qPCR) using the following primers: gyrB-1 (5′- TTGTCAGATGTAAACAACACGG-3′), gyrB-2 (5′- GTCCGTTATCCGTTACTTTAATCC-3′), Tn-F(5′- TTTATGGTA CCATTTCATTTTCCTGCTTTTTC), geh-1 (5′-GCAACCA TATTGTTATG ACCACC-3′), and saeR-1(5′- CAACCAGTTGAACAACTGTCG-3′). The primer pair gyrB-1/gyrB-2 was used to normalize DNA sample loading, while the primer pairs Tn-F/geh-1 and Tn-F/saeR-1 were used to assess changes of the *geh* and *saeR* transposon mutants in the final cultures. The DNA amplifications were performed with a 7900HT Fast Real Time PCR system (Applied Biosystems) using the following conditions: 94°C 30 sec, 55°C 30 sec, 72°C 30 sec, 40 cycles. The change of each strain was calculated with the following formula:
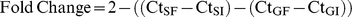
where Ct_SF_  =  Ct of test gene (i.e. either *geh* or *saeR*) in the final cultures; Ct_SI_  =  Ct of test gene in the initial mixed culture; Ct_GF_  =  Ct of *gyrB* in the final cultures; Ct_GI__ = _ Ct of *gyrB* in the initial mixed culture.

The enrichment index was calculated with the following formula:

Enrichment Index  =  (Fold Change of *saeR* mutant)/(Fold Change of *geh* mutant).

## Results

### The *ausA* transposon mutant ΦΝΞ-12202 does not produce aureusimines

We used the *ausA* transposon mutant ΦΝΞ-12202 from the phoenix library collection [21], which was kindly provided by Drs. Olaf Schneewind and Dominique Missiakas (University of Chicago), to test whether aureusimines are required for the production of virulence factors. The ΦΝΞ-12202 mutant strain has a transposon insertion 36 bp downstream of the putative ATG start codon of *ausA* (Fig. 2A). To eliminate any fortuitous mutations acquired during the mutagenesis process, the transposon insertion was transduced into strain Newman with the staphylococcal phage φ85. To confirm the transposon insertion mutation of *ausA*, we subjected the mutant chromosomal DNA to PCR-amplification with the primer pairs PM1/PM2 or PM1/PM3 (Fig. 2A). Since primer PM2 binds to the *bursa aurealis* transposon sequence used for construction of the Phoenix library, a PCR product with the primer pair PM1/PM2 can be amplified only from genomic DNA of the transposon mutant. On the other hand, since both PM1 and PM3 primers recognize *ausA* sequences, PM1/PM3 PCR products are expected to be amplified from genomic DNA of both the wild type and the transposon mutant strains, but with different sizes. The size of the product amplified from the mutant DNA is expected to be 3.2 kb larger than that from wild-type DNA. Accordingly, using primer pairs PM1 and PM2, and PM1 and PM3, PCR products with the expected sizes of approximately 800 bp, or 3.8 kb, respectively, were amplified using DNA from the transposon mutant (Fig. 2B). The latter PCR product with a molecular weight of 3.8 kb corresponds to the sum of the *ausA* (621 bp) and the transposon (3.2 kb) sequences. The PM1/PM2 PCR product obtained from the mutant strain DNA was sequenced, confirming the transposon insertion site (data not shown). To verify that the *ausA* mutant does not produce aureusimines, we compared the culture supernatants from the wild-type and *ausA* mutant strains by organic extraction and high performance liquid chromatography (HPLC) [15]. We chemically synthesized aureusimine A as standard (Fig. S1). While two peaks corresponding to aureusimines A and B were detected in the supernatant from the wild-type strain Newman, those peaks were not detected in the culture supernatant from the transposon mutant (Fig. 2C), thus confirming the result of Wyatt et al. that *ausA* encodes the dipeptide synthesis enzyme [15]. As expected, the elution time of the chemically synthesized aureusimine A corresponded to that of the first peak, interpreted as aureusimine A. These results demonstrate that the transposon mutant ΦΝΞ-12202 does not produce aureusimines and therefore represents an appropriate tool for the study of the role of aureusimine dipeptides.

### SaeRS is functional in the *ausA* transposon mutant ΦΝΞ-12202

**Figure 3:**
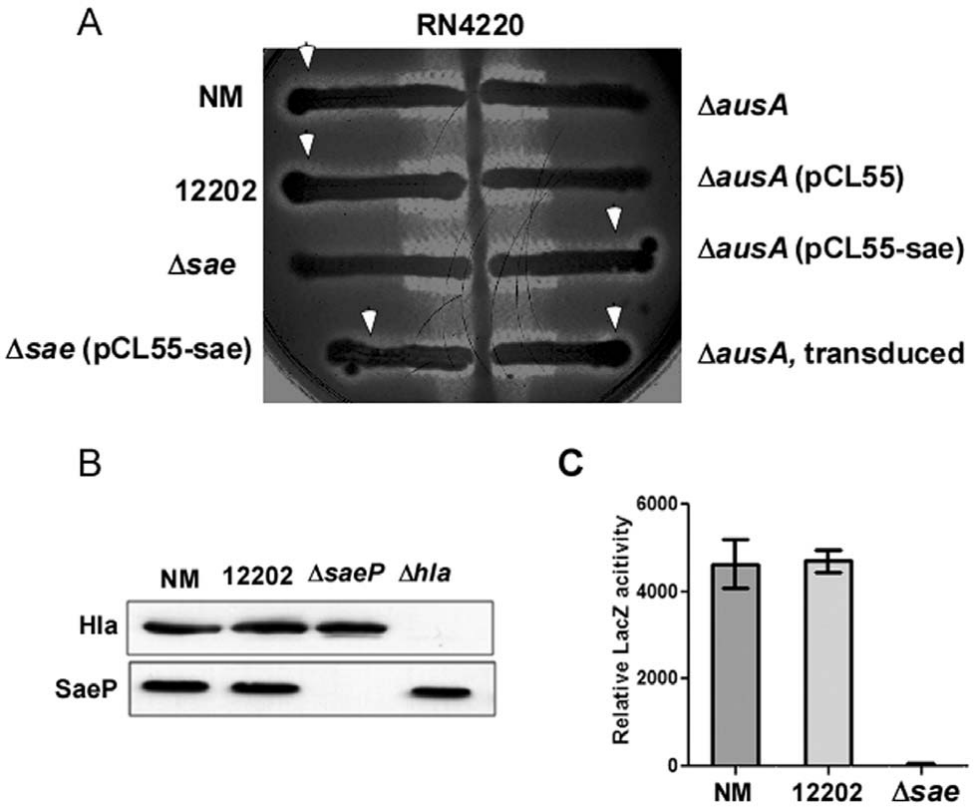
The SaeRS TCS is functional in the *ausA* transposon mutant ΦΝΞ-12202. **A**. Hemolysis patterns on sheep blood agar. The strains tested were streaked against the strain RN4220, which produces only beta-hemolysin. The white arrow indicates the hemolysis caused by alpha-hemolysin. The enhanced hemolysis at the junctions of strain RN4220 and the tested strains is due to delta-hemolysin production originating from the test strains. NM, wild type; 12202, transposon insertion mutant ΦΝΞ-12202; Δ*sae*, a deletion mutant of the *sae* operon; Δ*ausA*, original *ausA* deletion mutant; pCL55-sae, pCL55 plasmid containing the entire *sae* operon. **B**. Western blot analysis for alpha-hemolysis (Hla) and SaeP protein. NM, wild type; 12202, ΦΝΞ-12202; Δ*saeP*, a transposon insertion mutant of *saeP*; Δ*hla*, a transposon insertion mutant of *hla*, the gene encoding alpha-hemolysin. **C.** LacZ assay for P1 promoter activity. Mid-log phase cells were used to measure LacZ activity from P1-*lacZ* fusion in strains Newman (NM), ΦΝΞ-12202, and the *sae* deletion mutant (Δ*sae).* Bar graphs depict the mean ± standard deviation for the relative LacZ activity of each indicated strain.

Next, we tested whether the aureusimine dipeptides are required for SaeRS signaling using the mutant strain ΦΝΞ-12202. First, because SaeRS is critical for alpha-hemolysin production, we examined the hemolysis pattern of the mutant on blood agar. The tested strains were streaked against the strain RN4220, which produces only beta-hemolysin [22]. To our surprise, the hemolysis pattern of the *ausA* transposon mutant was identical to that of wild type (see the white arrow heads in Fig. 3A), whereas the *sae* deletion mutant (Δ*sae* in Fig. 3A) showed a clear defect in hemolysis. Furthermore, wild-type level hemolysis was restored by insertion of the complementation plasmid pCL55-sae [Δ*sae*(pCL55-sae) in Fig. 3A] in the *sae* deletion mutant. These results strongly indicate that *ausA* is not involved in controlling expression of alpha-hemolysin, which is at variance with the results obtained by Wyatt et al. [15].

To further test whether the *ausA* mutant produces alpha-hemolysin, we used Western blot analysis. In addition, we examined expression of SaeP, another well-known gene product of the *sae* operon. Consistent with the hemolysis patterns on blood agar, the *ausA* transposon mutant ΦΝΞ-12202 produced both alpha-hemolysin and SaeP at the wild-type level (Fig. 3B), suggesting that SaeRS signaling is intact in the *ausA* mutant. When transcription from the P1 promoter of the *sae* operon was examined using a P1-*lacZ* fusion, no significant difference was observed between the wild type and the mutant strain (Fig. 3C), further confirming the normal functionality of SaeRS in the *ausA* transposon mutant. These data demonstrate that *ausA* and the biosynthetic products of the *ausA* locus, the aureusimines, are not required for SaeRS signaling.

### Aureusimines do not contribute to staphylococcal virulence

**Figure 4:**
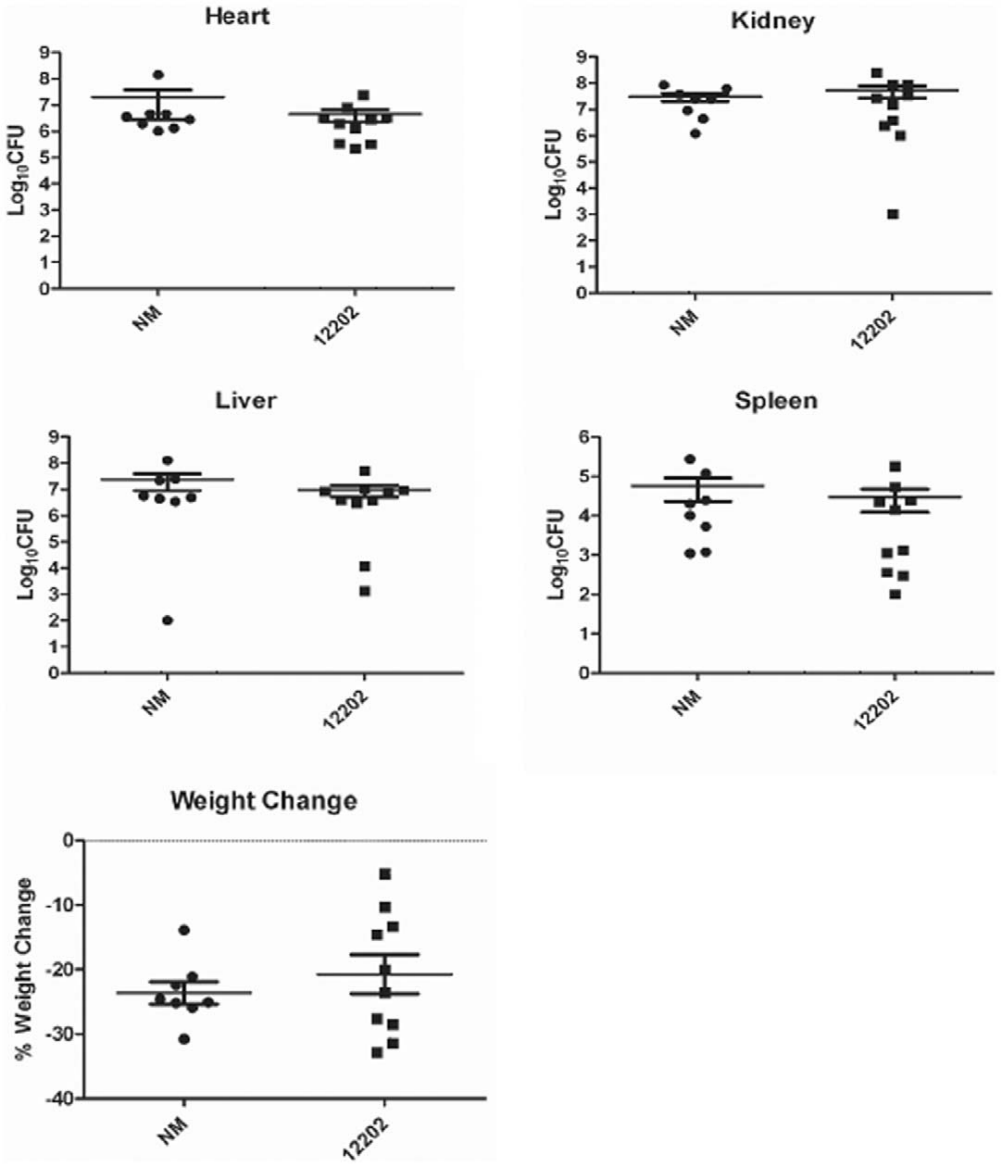
Aureusimines do not contribute to staphylococcal virulence. The test strains (1×10^7^ cfu) were administered into 10 mice per strain via retro-orbital injection; then, four days later, after measuring weight losses, organs were harvested and bacterial cfu in the organs was determined. In the graph, each dot represents data from one mouse. Mean and standard deviation are indicated.

Given that our findings demonstrate that aureusimines are not required for SaeRS signaling and SaeRS-dependent alpha-hemolysin production, we further tested the contribution of aureusimines to bacterial virulence. Equal numbers of cells of the wild-type Newman and transposon mutant ΦΝΞ-12202 strains were injected into mice; then effects on disease development were analyzed in the same way as in the Wyatt et al. study [15]. Bacterial numbers in all tested organs were not significantly different between mice infected with the wild-type or mutant strains (Fig. 4). In addition, no significant difference was observed in weight loss, showing that aureusimines are not required for staphylococcal virulence in this murine infection model.

### The original *ausA* deletion mutant contains an additional mutation in *saeS*

Since we failed to observe a role of aureusimines in hemolysin production or in-vivo virulence, we suspected that the original *ausA* deletion mutant might have contained additional mutation(s), especially in the *saeRS* genes. Therefore, we examined the *saeRS* region of the original mutant used by Wyatt et al. by DNA sequencing, which revealed that the original mutant has an 83-nt duplication in *saeS* (Fig. 1). Furthermore, the hemolysis defect of the original *ausA* mutant on blood agar was fully complemented by the plasmid carrying the *sae* operon [Δ*ausA*(pCL-sae) in Fig. 3A]. In addition, when transduced into strain Newman, the resulting *ausA* deletion mutant showed the wild-type hemolysis pattern (‘Δ*ausA*, transduced’ in Fig. 3A). These results demonstrate that the virulence defects of the *ausA* deletion mutant in the Wyatt et al. study were caused by the inadvertent mutation in *saeS* that we discovered.

### The *sae* deletion mutant is more resistant to certain antibiotics

**Figure 5:**
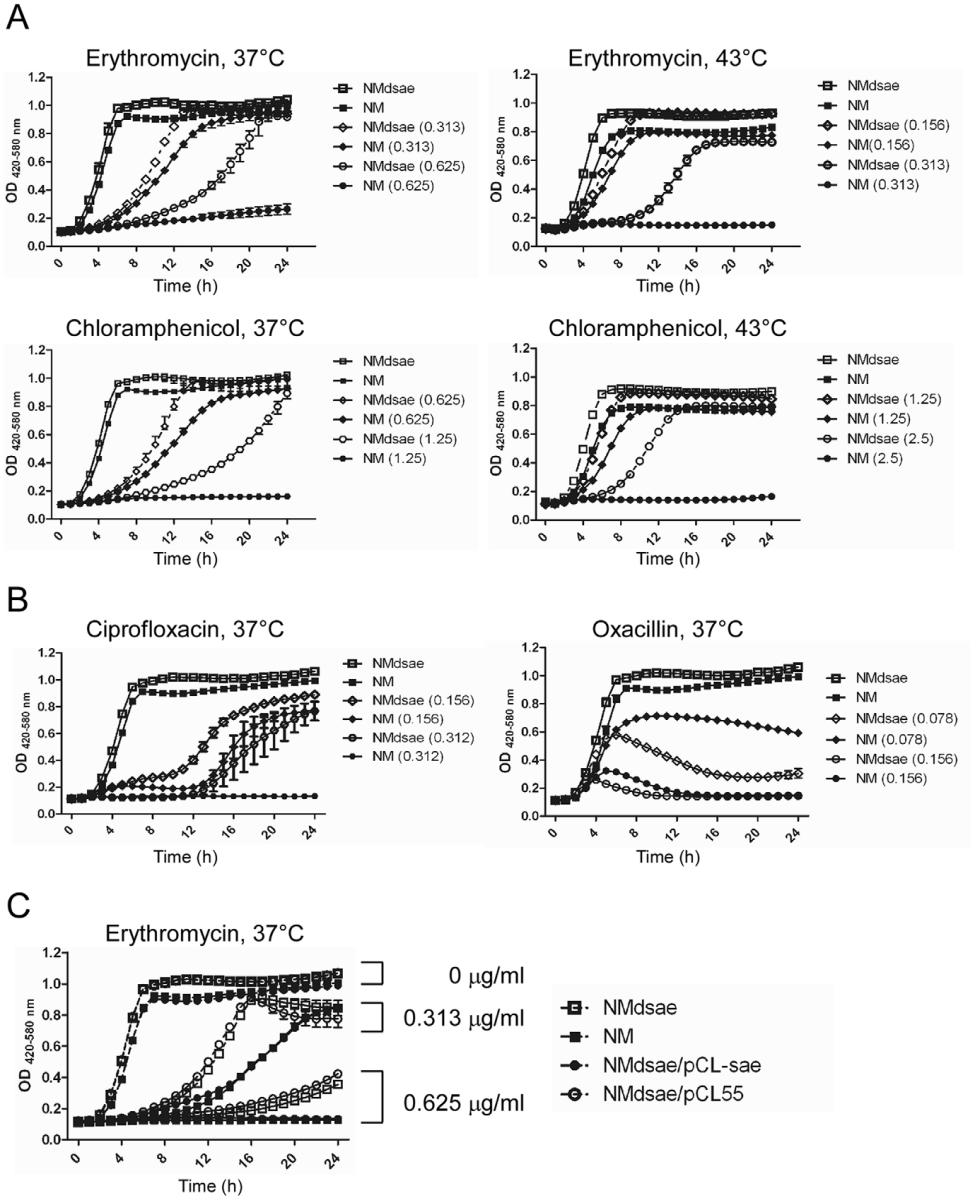
The *sae* deletion mutant is more resistant to certain antibiotics. **A.** Effect of the *sae* deletion mutation on resistance to chloramphenicol and erythromycin at 37°C or 43°C. Cells were inoculated in 200 µl TSB; then the growth of the cells was measured with Bioscreen C (Growth Curves USA) for 24 h. The antibiotics and growth temperatures are shown above each graph. The concentration (µg/ml) of antibiotics is shown in parentheses. **B.** Effect of the *sae* deletion mutation on resistance to ciprofloxacin and oxacillin at 37°C. **C.** Complementation test for the *sae* deletion mutant. The used erythromycin concentration is indicated to the right of the graph. pCL55, an integration vector; pCL55-sae, pCL55 containing the entire *sae* operon.

Next, we examined whether acquisition of the additional mutation in *saeS* may have resulted from conditions during mutagenesis that selected for the mutant. Since mutagenesis often involves growing *S. aureus* cells in the presence of antibiotics such as chloramphenicol and erythromycin, if mutations in the *saeRS* system render cells more resistant to these antibiotics, s*aeRS* mutants would be enriched during the mutagenesis process. We tested this hypothesis by comparing the growth of wild-type Newman and *sae* deletion mutant strains in the presence of various antibiotics. At both 37°C and 43°C, the *sae* deletion mutant showed higher resistance to chloramphenicol and erythromycin (Fig. 5A). Although to a lesser extent, the mutant was also more resistant to spectinomycin at 43°C and kanamycin at 37°C (data not shown). Furthermore, because all of the antibiotics tested are protein synthesis inhibitors, we investigated whether the *sae* deletion mutant is also more resistant to other classes of antibiotics. At 37°C, the *sae-*deletion mutant showed a low level of resistance to ciprofloxacin, a DNA gyrase inhibitor (Fig. 5B). However, the mutant was more sensitive to cell lysis caused by oxacillin, a cell-wall synthesis inhibitor. Both wild-type and the *sae* deletion mutant strains were equally sensitive to rifampicin, an RNA synthesis inhibitor, and to tetracycline, another protein synthesis inhibitor (data not shown). When complemented by the plasmid pCL55-sae, the resistance to erythromycin was abolished (Fig. 5C), confirming that increased resistance was due to the deletion of the *sae* operon.

### A *sae* mutant has a growth advantage at high temperature, aeration, and presence of erythromycin

The moderate resistance to erythromycin and chloramphenicol strongly suggests that *sae* mutants can be enriched during mutagenesis when employing antibiotics. However, since in a typical mutagenesis process (e.g., deletion or transposon insertion) the target strains already have plasmid-conferred antibiotic resistance, the comparison between the wild-type and *sae* deletion mutant strains shown in Fig. 5 might not be directly relevant to the mutagenesis process. To examine whether *sae* mutants have a growth advantage and can be enriched even with a strain that is already resistant to antibiotics, we used two transposon mutants, NM::*geh* and NM::*saeR*. Due to the presence of an erythromycin resistance gene in the transposon, both strains are fully resistant to erythromycin (i.e., minimal inhibitory concentration >100 µg/ml). NM::*geh* has a transposon insertion in *geh* (glycerol ester hydrolase) while NM::*saeR* has a transposon insertion in *saeR*. Since *geh* is already disrupted by the lysogenic phage φNM4 [23] in strain Newman, further disruption by the transposon insertion in *geh* is not expected to affect bacterial gene expression, physiology, or virulence.

**Figure 6:**
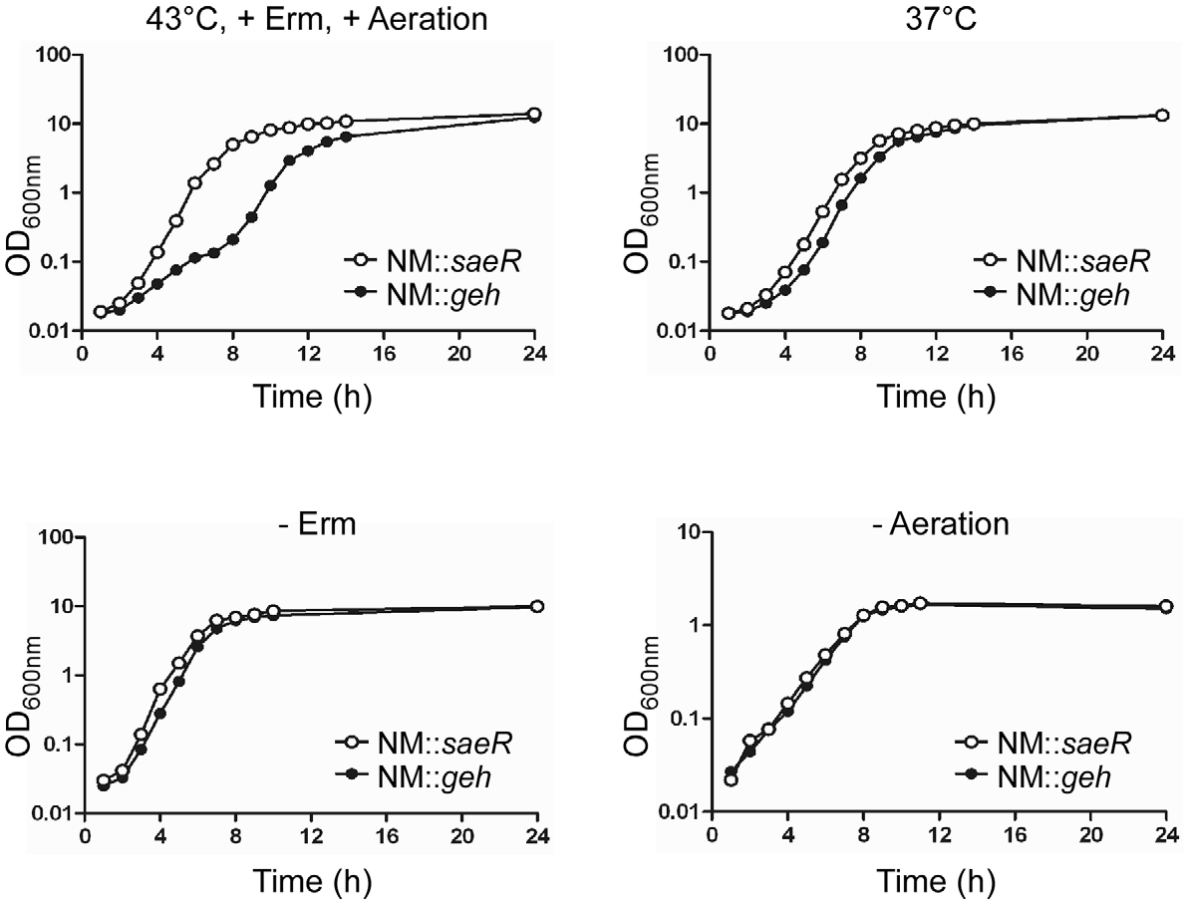
An *sae* mutant has a growth advantage at high temperature, aeration, and presence of erythromycin. The transposon mutants of *saeR* and *geh* (glycerol ester hydrolase) were grown under various conditions and the effects of three environmental factors (temperature, the presence of erythromycin, and aeration) on the growth pattern of the mutants were examined. For clarity, only the altered environmental factor is indicated above the graph being compared. In the test, erythromycin was added at 10 µg/ml and aeration was achieved by shaking at 250 rpm. Erm; erythromycin.

The mutants were incubated in TSB containing 10 µg/ml of erythromycin (TSB_erm10_) overnight at 37°C; then equal numbers of cells were inoculated into TSB_erm10_ and incubated with aeration (250 rpm) at either 37°C or 43°C. The growth advantage was pronounced at 43°C, at which temperature NM::*geh* showed a diphasic growth pattern (Fig. 6). The doubling time was 37 min for NM::*saeR* while it was 113 min (4 hr–8 hr) or 44 min (9 hr–12 hr) for NM::*geh*. On the other hand, at 37°C, although NM::*geh* showed a little longer lag phase, the growth rate at mid-log phase was almost identical (doubling time for NM::*saeR*, 41.3 min; NM::*geh*, 40.8 min). Interestingly, when we repeated the growth test at 43°C in the absence of either erythromycin or aeration, the pronounced growth advantage of NM::*saeR* over NM::*geh* mutant was greatly diminished (Fig. 6, bottom panel). Without erythromycin, the doubling time for NM::*saeR* was 30.6 min, while it was 38.9 min for NM::*geh*. These results suggest that high temperature, presence of erythromycin, and aeration contribute to the growth advantage of the *sae* mutant.

### *Sae* mutants can be enriched under typical staphylococcal mutagenesis conditions

**Figure 7:**
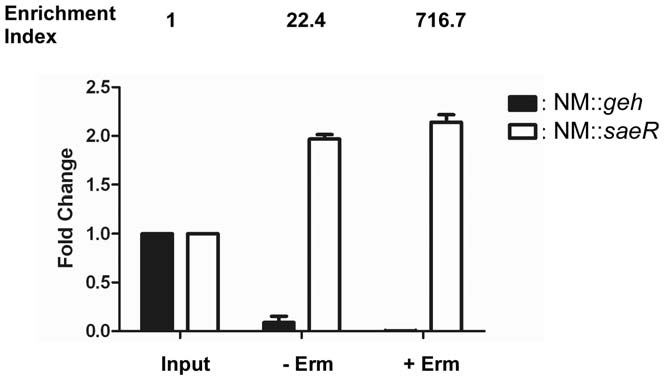
*Sae* mutants can be enriched under typical staphylococcal mutagenesis conditions. Transposon insertion mutants of *geh* (glycerol ester hydrolase) and *saeR* were used in this test. Equal numbers of the two mutants were mixed and grown either in the absence or in the presence of erythromycin (10 µg/ml) with shaking for 16 h at 43°C. Chromosomal DNA was purified from each culture and the fold change of each strain was calculated by quantitative real time PCR (qPCR) as described in Materials and Methods. Bar graphs indicate the fold change of each strain. Data are presented as the mean ± standard deviation from three independent experiments. Input, the mixed culture used for inoculation; - Erm, culture grown in the absence of erythromycin; + Erm, culture grown in the presence of erythromycin.

In a typical staphylococcal mutagenesis procedure, the target cells are incubated at 43C° in the presence of an antibiotic and aeration, which according to our results favors growth of *sae* mutants (Fig. 6). To examine the extent of the enrichment of the *sae* mutant under those growth conditions, an equal number of NM::*geh* and NM::*saeR* cells were combined and incubated for 16 h at 43°C with aeration in the presence of erythromycin (10 µg/ml). For comparison, we also grew the mixed cells in the absence of erythromycin. To assess the relative ratio of the mutants in the resulting cultures, we purified chromosomal DNA and performed quantitative real-time PCR (qPCR), amplifying parts of the transposon sequence, and *geh* or *saeR*. While, in the absence of erythromycin, the NM::*saeR* strain was enriched approximately 20 fold, the enrichment was increased to approximately 700 fold in the presence of erythromycin (Fig. 7). These results demonstrate that *sae* mutants can be enriched to a great extent under conditions typically employed for staphylococcal mutagenesis.

## Discussion

The SaeRS TCS is an important signaling system controlling the production of multiple virulence factors and *in vivo* survival of *S. aureus* [4], [5], [8]. However, how the signaling system is activated remains unknown. Although results from the Wyatt et al. study raised the possibility that the dipeptide aureusimines could be the activation signal for SaeRS [15], we here demonstrate that the results obtained by Wyatt et al., indicating an involvement of the dipeptides in SaeRS signaling, were due to an inadvertent mutation in *saeS*. We suspect that the acquisition of the *saeS* mutation was facilitated by the increased resistance of *sae* mutants to environmental stresses such as high temperature, aeration, and the presence of erythromycin, the antibiotic used for the generation of a single cross-over during the deletion of *ausA* [15].

The *sae* deletion mutant showed a moderate level of resistance not only to erythromycin but also to chloramphenicol, another antibiotic widely used in staphylococcal research (Fig. 5A). Both antibiotics inhibit protein synthesis by binding to the 50S subunit of bacterial ribosome [24], [25]. Although the exact steps inhibited by these two antibiotics are different, the binding sites of the antibiotics overlap [25]. Therefore, it is possible that the same change caused by the *saeRS* TCS mutation is responsible for the elevated resistance to the antibiotics. However, the nature of that change is not yet clear. One can hypothesize that some genes in the *sae* regulon might be directly involved in the protein translation process and can affect ribosomal sensitivity to the antibiotics. Among the genes that contain SaeR-binding sequences in the upstream region, six are involved in protein translation: peptide chain release factor-2 (*prf*), 30S ribosomal proteins S20 and S21, alanyl-tRNA synthetase, glutamyl-tRNA synthetase, and histidyl-tRNA synthetase [16]. It remains to be determined if these or other factors contribute to the resistance to certain antibiotics in the *saeRS* mutants.

Given that the synthesis of many exoproteins is decreased in *sae* mutants, it is not surprising to observe a measurable growth advantage of the *saeR* mutant over the *geh* mutant (Fig. 6). Intriguingly, the growth advantage of *sae* mutants was greatly enhanced by the combination of three environmental stresses: high temperature, the presence of erythromycin, and aeration. A recent study also showed that *sae* mutants are 10 times more resistant to the oxidative stresses imposed by H_2_O_2_ [26], which is known to activate the SaeRS TCS [11]. These results raise the question of why *sae* mutants are more resistant to those stresses. It is possible that certain gene products of the *sae* regulon might exacerbate or amplify the growth inhibitory effects of those stresses. However, because not all genes belonging to the *sae* regulon are yet verified experimentally, it is difficult at this point to identify candidates for the gene products responsible for the exacerbated growth inhibition.

Interestingly, unlike *sae* mutants, mutants of the *agr* quorum-sensing system, another TCS important for staphylococcal virulence, seem to be enriched in the absence of environmental stresses [27]. In fact, due to the genetic instability of *agr*, global regulatory roles have been bestowed incorrectly to several genes such as *xpr*, *svrA*, *traP*, and *lukSF-PV*. The genetic locus *xpr* (more correctly the mutation Δ 1058::Tn*551*) was identified, in a Tn*551* mutagenesis, to encode a global regulator required for the expression of lipase, alpha-toxin, delta-toxin, protease, and nuclease [28]. However, later, all of the expression defects of the *xpr* mutant were found to be caused by an additional mutation in *agrC*, the gene encoding *agr* sensor kinase [29]. The gene *svrA* was identified in a signature-tagged mutagenesis (STM) screen as a positive regulator for *agr* expression because the *svrA* mutation abolished expression of *agr* [30], [31]. But the absence of *agr* expression in the *svrA* mutant was later attributed to a frame-shift mutation in *agrC* [32]. Although the *traP* gene was initially reported to activate the *agr* system via a pathway distinct from the autoinducing peptide (AIP) pathway [33], Adhikari et al showed that the *traP* mutant contains a stop codon in *agrA*, the gene encoding the response regulator, which, in turn, explains the lack of *agr* activity in the *traP* mutant [22]. Finally, a PVL phage strain of *S. aureus* producing Panton-Valentine Leukocidin (PVL), a pore-forming toxin encoded by *lukSF-PV*, was reported to exhibit an *agr-*negative phenotype (i.e., reduced expression of multiple exoproteins and enhanced expression of surface proteins such as SdrD and Spa) [34]. However, Villaruz et al. demonstrated that the PVL phage strain used in the original study carried a point mutation at the AgrA binding site in *agr* P2 promoter; therefore, the *agr*-negative phenotype of the PVL phage strain was caused by the point mutation in the *agr* P2 promoter, not by expression of PVL [35]. Together with our report on the *saeS* mutation in the *ausA* mutant, these examples clearly show that great care should be taken during genetic manipulation of *S. aureus* in order to prevent acquisition of secondary mutations, especially in *saeRS* and *agr,* and appropriate genetic complementation experiments used.

Currently, the use of antibiotics during mutagenesis of staphylococcal genes is inevitable. Since *sae* mutants show elevated resistance to chloramphenicol, erythromycin, and, to a lesser extent, kanamycin, spectinomycin, and ciprofloxacin, when one of these antibiotics is used, it would be prudent to confirm that the resulting mutant does not contain an inadvertent mutation in the *sae* operon. For confirmation, a convenient method is using a hemolysis assay on blood agar. In particular, if the test strain is streaked on sheep blood agar against RN4220 as shown in Fig. 3A [22], the enhancement of β-hemolysis by delta-hemolysin at the strain junction can also reveal the functionality of the *agr* quorum-sensing system. This is of particular importance because *agr* mutants can arise spontaneously in the laboratory as well as in clinical settings [22], [27], [29], [32], [35], [36], [37], [38]. Since the *sae* mutants do not seem to be more resistant to tetracycline, if a choice is given, usage of tetracycline can minimize the enrichment of *sae* mutants.

Since our results showed that aureusimines do not play a role in *sae*-mediated virulence factor production or virulence in animal models, it remains to be determined what roles these dipeptides play in *S. aureus.* Intriguingly, the upstream sequence of *ausA* contains an imperfect SaeR binding sequence (ATTAAGAATTTGTTAA ), indicating that the expression of *ausAB* genes might be regulated by *saeRS* TCS [16]. Certainly, more work is required to identify the role(s) of these dipeptides in *S. aureus*.

## Supporting Information

Figure S1 **Chemical synthesis of aureusimine A.** For each step, reagents and reaction conditions are presented with yields. DCC, N,N'-Dicyclohexylcarbodiimide; HOBT, Hydroxybenzotriazole; DIPEA, Diisopropylethylamine; DIBAL, Diisobutylaluminium hydride; THF, tetrahydrofuran; DCM, dimethylene chloride; TEA, triethylamine; RT, room temperature.
(DOC)

## References

[1] Archer GL (1998). *Staphylococcus aureus:* a well-armed pathogen.. Clin Infect Dis.

[2] Lowy FD (1998). *Staphylococcus aureus* infections.. N Engl J Med.

[3] Novick RP (2003). Autoinduction and signal transduction in the regulation of staphylococcal virulence.. Mol Microbiol.

[4] Giraudo AT, Calzolari A, Cataldi AA, Bogni C, Nagel R (1999). The *sae* locus of *Staphylococcus aureus* encodes a two-component regulatory system.. FEMS Microbiol Lett.

[5] Giraudo AT, Cheung AL, Nagel R (1997). The *sae* locus of *Staphylococcus aureus* controls exoprotein synthesis at the transcriptional level.. Arch Microbiol.

[6] Giraudo AT, Raspanti CG, Calzolari A, Nagel R (1994). Characterization of a Tn551-mutant of *Staphylococcus aureus* defective in the production of several exoproteins.. Can J Microbiol.

[7] Liang X, Yu C, Sun J, Liu H, Landwehr C (2006). Inactivation of a two-component signal transduction system, SaeRS, eliminates adherence and attenuates virulence of *Staphylococcus aureus*.. Infect Immun.

[8] Voyich JM, Vuong C, Dewald M, Nygaard TK, Kocianova S (2009). The SaeR/S Gene Regulatory System Is Essential for Innate Immune Evasion by *Staphylococcus aureus*.. J Infect Dis.

[9] Xiong YQ, Willard J, Yeaman MR, Cheung AL, Bayer AS (2006). Regulation of *Staphylococcus aureus* alpha-toxin gene (*hla*) expression by *agr, sarA*, and *sae* in vitro and in experimental infective endocarditis.. J Infect Dis.

[10] Adhikari RP, Novick RP (2008). Regulatory organization of the staphylococcal *sae* locus.. Microbiology.

[11] Geiger T, Goerke C, Mainiero M, Kraus D, Wolz C (2008). The Virulence Regulator Sae of *Staphylococcus aureus*: Promoter Activities and Response to Phagocytosis-Related Signals.. J Bacteriol.

[12] Li D, Cheung A (2008). The repression of *hla* by *rot* is dependent on *sae* in *Staphylococcus aureus*.. Infect Immun.

[13] Giraudo AT, Mansilla C, Chan A, Raspanti C, Nagel R (2003). Studies on the expression of regulatory locus *sae* in *Staphylococcus aureus*.. Curr Microbiol.

[14] Goerke C, Fluckiger U, Steinhuber A, Bisanzio V, Ulrich M (2005). Role of *Staphylococcus aureus* global regulators *sae* and sigmaB in virulence gene expression during device-related infection.. Infect Immun.

[15] Wyatt MA, Wang W, Roux CM, Beasley FC, Heinrichs DE (2010). *Staphylococcus aureus* Nonribosomal Peptide Secondary Metabolites Regulate Virulence.. Science.

[16] Sun F, Li C, Jeong D, Sohn C, He C (2010). In the *Staphylococcus aureus* two-component system *sae*, the response regulator SaeR binds to a direct repeat sequence and DNA binding requires phosphorylation by the sensor kinase SaeS.. J Bacteriol.

[17] Bae T, Schneewind O (2006). Allelic replacement in *Staphylococcus aureus* with inducible counter-selection.. Plasmid.

[18] Zeng Y, Li Q, Hanzlik RP, Aube J (2005). Synthesis of a small library of diketopiperazines as potential inhibitors of calpain.. Bioorg Med Chem Lett.

[19] Lee CY, Buranen SL, Ye ZH (1991). Construction of single-copy integration vectors for *Staphylococcus aureus*.. Gene.

[20] Li C, Sun F, Cho H, Yelavarthi V, Sohn C (2010). CcpA mediates proline auxotrophy and is required for *Staphylococcus aureus* pathogenesis.. J Bacteriol.

[21] Bae T, Banger AK, Wallace A, Glass EM, Aslund F (2004). *Staphylococcus aureus* virulence genes identified by bursa aurealis mutagenesis and nematode killing.. Proc Natl Acad Sci U S A.

[22] Adhikari RP, Arvidson S, Novick RP (2007). A nonsense mutation in *agrA* accounts for the defect in *agr* expression and the avirulence of *Staphylococcus aureus* 8325-4 *traP*::kan.. Infect Immun.

[23] Baba T, Bae T, Schneewind O, Takeuchi F, Hiramatsu K (2008). Genome Sequence of *Staphylococcus aureus* Strain Newman and Comparative Analysis of Staphylococcal Genomes: Polymorphism and Evolution of Two Major Pathogenicity Islands.. J Bacteriol.

[24] Lovmar M, Nilsson K, Lukk E, Vimberg V, Tenson T (2009). Erythromycin resistance by L4/L22 mutations and resistance masking by drug efflux pump deficiency.. EMBO J.

[25] Moazed D, Noller HF (1987). Chloramphenicol, erythromycin, carbomycin and vernamycin B protect overlapping sites in the peptidyl transferase region of 23S ribosomal RNA.. Biochimie.

[26] Johnson M, Sengupta M, Purves J, Tarrant E, Williams PH (2010). Fur is required for the activation of virulence gene expression through the induction of the *sae* regulatory system in *Staphylococcus aureus*.. Int J Med Microbiol in press.

[27] Somerville GA, Beres SB, Fitzgerald JR, DeLeo FR, Cole RL (2002). In vitro serial passage of *Staphylococcus aureus*: changes in physiology, virulence factor production, and *agr* nucleotide sequence.. J Bacteriol.

[28] Smeltzer MS, Hart ME, Iandolo JJ (1993). Phenotypic characterization of *xpr*, a global regulator of extracellular virulence factors in *Staphylococcus aureus*.. Infect Immun.

[29] McNamara PJ, Iandolo JJ (1998). Genetic instability of the global regulator *agr* explains the phenotype of the *xpr* mutation in *Staphylococcus aureus* KSI9051.. J Bacteriol.

[30] Garvis S, Mei JM, Ruiz-Albert J, Holden DW (2002). *Staphylococcus aureus svrA*: a gene required for virulence and expression of the *agr* locus.. Microbiology.

[31] Mei JM, Nourbakhsh F, Ford CW, Holden DW (1997). Identification of *Staphylococcus aureus* virulence genes in a murine model of bacteraemia using signature-tagged mutagenesis.. Mol Microbiol.

[32] Chen J, Novick RP (2007). *svrA*, a multi-drug exporter, does not control *agr*.. Microbiology.

[33] Balaban N, Goldkorn T, Gov Y, Hirshberg M, Koyfman N (2001). Regulation of *Staphylococcus aureus* pathogenesis via target of RNAIII-activating Protein (TRAP).. J Biol Chem.

[34] Labandeira-Rey M, Couzon F, Boisset S, Brown EL, Bes M (2007). *Staphylococcus aureus* Panton Valentine Leukocidin Causes Necrotizing Pneumonia.. Science.

[35] Villaruz AE, Wardenburg JB, Khan BA, Whitney AR, Sturdevant DE (2009). A Point Mutation in the *agr* Locus rather than Expression of the Panton-Valentine Leukocidin Caused Previously Reported Phenotypes in *Staphylococcus aureus* Pneumonia and Gene Regulation.. J Infect Dis.

[36] Shopsin B, Drlica-Wagner A, Mathema B, Adhikari RP, Kreiswirth BN (2008). Prevalence of *agr* dysfunction among colonizing *Staphylococcus aureus* strains.. J Infect Dis.

[37] Traber K, Novick R (2006). A slipped-mispairing mutation in AgrA of laboratory strains and clinical isolates results in delayed activation of *agr* and failure to translate delta- and alpha-haemolysins.. Mol Microbiol.

[38] Fowler VG, Sakoulas G, McIntyre LM, Meka VG, Arbeit RD (2004). Persistent bacteremia due to methicillin-resistant *Staphylococcus aureus* infection is associated with *agr* dysfunction and low-level in vitro resistance to thrombin-induced platelet microbicidal protein.. J Infect Dis.

[39] Kreiswirth BN, Lofdahl S, Betley MJ, O'Reilly M, Schlievert PM (1983). The toxic shock syndrome exotoxin structural gene is not detectably transmitted by a prophage.. Nature.

[40] Duthie ES, Lorenz LL (1952). Staphylococcal coagulase; mode of action and antigenicity.. J Gen Microbiol.

