# Supplementary material for: Aureusimines in Staphylococcus aureus Are Not Involved in Virulence
Source: PLoS One. 2010 Dec 29;5(12):e15703. doi: 10.1371/journal.pone.0015703 (PMC3012096; doi:10.1371/journal.pone.0015703)
Supplement: Figure S1 — Chemical synthesis of aureusimine A. For each step, reagents and reaction conditions are presented with yields. DCC, N,N'-Dicyclohexylcarbodiimide; HOBT, Hydroxybenzotriazole; DIPEA, Diisopropylethylamine; DIBAL, Diisobutylaluminium hydride; THF, tetrahydrofuran; DCM, dimethylene chloride; TEA, triethylamine; RT, room temperature. (DOC) [file pone.0015703.s001.doc]

The overall synthesis scheme is shown below.

**
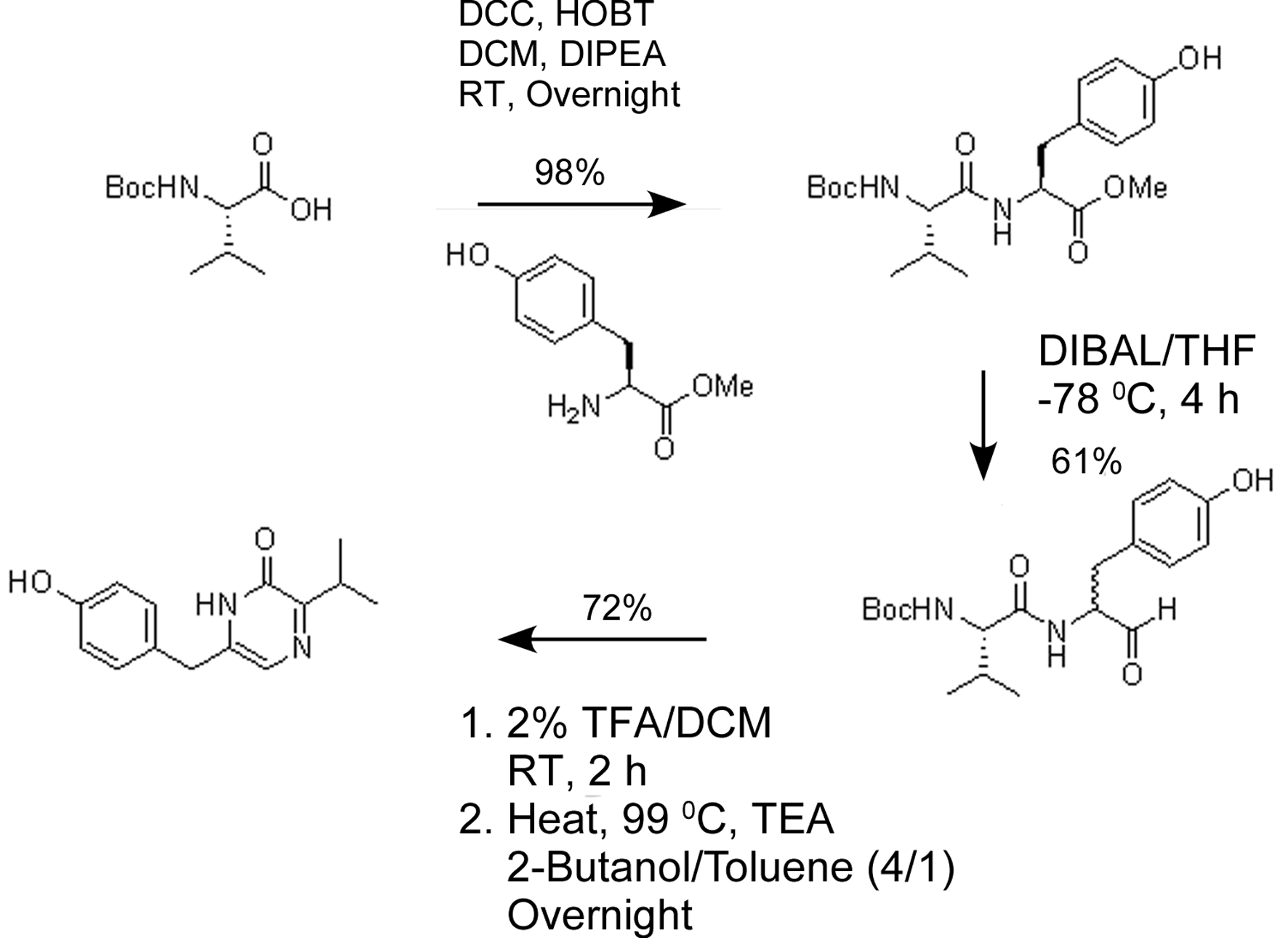
**

1. **Boc-Val-Tyr-OMe synthesis**

To a 100 ml round-bottom flask was added Boc-protected Valine (1.5 g, 6.9 mmol), L-Tyrosine methyl ester (1.6 g, 6.9 mmol), N,N'-Dicyclohexylcarbodiimide (DCC, 1.5 g, 6.9 mmol), Hydroxybenzotriazole (HOBT, 0.98 g, 6.9 mmol), N,N-Diisopropylethylamine (DIPEA, 2.0 g, 13.8 mmol) and 35 ml dimethylene chloride (DCM). The reaction was stirred at room temperature overnight. The reaction was monitored by thin layer chromatography (TLC) until it proceeded completely. The resulting mixture was poured into 35 ml of water and extracted by ethyl acetate (40 ml ×3). The organic layer was further washed with 10% citric acid (80 ml), water (80 ml), 5% NaHCO_3_ (80 ml) and brine (80 ml), dried with Na_2_SO_4_ (s) and evaporated. The dipeptide product was directly used for the next step without additional purification. MS calcd. 294.2 (M-C4H9CO2), 338.2 (M-C4H9), found 295.1 (M-C4H9CO2+H), 339.1 (M-C4H9+H); H-NMR (deuterated DMSO) δ (integration, multiplicity, *J* in Hz) 0.79 (6-H, doublet, 0.30), 1.39 (9-H, singlet), 1.87 (1-H, m, 0.17), 2.85 (2-H, m, 0.015), 3.56 (3-H, singlet), 3.80 (1-H, m), 4.41 (1-H,m), 6.57 (1-H,doublet, 0.023) 6.65 (2-H, doublet, 0.021), 6.99 (2-H, doublet, 0.021), 8.177 (1-H, doublet, 0.018), 9.21 (1-H, singlet).

**Boc-Val-Tyr-CHO (2)**

Boc-Val-Tyr-OMe (0.82 g, 1.9 mmol) was dissolved in dry tetrahydrofuran (THF, 10 ml) and cooled down to -78°C in dry ice-acetone bath. The solution was degassed with N_2_. Diisobutylaluminium hydride (DIBAL, 1.5 M hexane solution, 3.2 ml) was added dropwise into the ester solution while stirring. The reaction was maintained at -78°C for 4 h and was quenched by ethyl acetate (5 ml). After 30 min, the reaction was raised to room temperature and treated with saturated potassium tartrate (10 ml) for another 30 min. The mixture was extracted with ethyl acetate (15 ml ×3), washed with brine (50 ml), dried with Na_2_SO_4_ and evaporated. The aldehyde product was briefly isolated by silica gel column (EA/Hex=1/1) and directly used for the next step.

**Aureusimine A (3)**

Boc-Val-Tyr-CHO (1 mmol) was dissolved in dimethylene chloride (DCM, 5 ml) containing 2% trifluoroacetic acid (TFA). The reaction was stirred at room temperature for 2 h and monitored by TLC. After the reaction was complete, the solvent was evaporated and the resulting crude product was dissolved in the solvent consisting of 2-butanol (1 ml), toluene (0.25 ml) and triethylamine (TEA) (1 mmol, 140 μl). The solution was heated at 99^o^C overnight. Aliquots of 0.25 ml of the resulting mixture were directly applied to HPLC purification as described previously. The retention time of aureusimine A was 19 min. The product was confirmed by MS, H-NMR and UV-Vis. MS calcd. 244.1, found 245.1 (M+H); H-NMR (deuterated DMSO) δ (integration, multiplicity, *J* in Hz)

1.09 (6-H, doublet), 3.22 (1-H, septet, 0.017), 3.61 (2-H, singlet), 6.69 (2-H, doublet, 0.016), 7.05 (1-H, singlet), 7.10 (2-H, doublet, 0.021), 9.30 (1-H, singlet), 12.15 (1-H, singlet); UV-Vis 324 nm.
